# Detection of sudomotor alterations evaluated by Sudoscan in patients with recently diagnosed type 2 diabetes

**DOI:** 10.1136/bmjdrc-2022-003005

**Published:** 2022-12-15

**Authors:** Ana Cristina García-Ulloa, Paloma Almeda-Valdes, Teresa Enedina Cuatecontzi-Xochitiotzi, Jorge Alberto Ramírez-García, Michelle Díaz-Pineda, Fernanda Garnica-Carrillo, Alejandra González-Duarte, K M Venkat Narayan, Carlos Alberto Aguilar-Salinas, Sergio Hernández-Jiménez

**Affiliations:** 1 Centro de Atención Integral del Paciente con Diabetes, Instituto Nacional de Ciencias Medicas y Nutricion Salvador Zubiran Departamento Endocrinologia y Metabolismo, Tlalpan, Mexico; 2 Endocrinology and Metabolism, Instituto Nacional de Ciencias Medicas y Nutricion Salvador Zubiran, Mexico City, Mexico; 3 Departamento de Neurología, Instituto Nacional de Ciencias Medicas y Nutricion Salvador Zubiran Departamento Endocrinologia y Metabolismo, Tlalpan, Mexico; 4 Rollins School of Public Health, Atlanta, Georgia, USA; 5 Dirección de Nutrición, Instituto Nacional de Ciencias Médicas y Nutrición Salvador Zubirán, Tlalpan, Mexico

**Keywords:** Normal, Foot, Diabetic Neuropathies, Diabetes Mellitus, Type 2

## Abstract

**Introduction:**

Diabetic peripheral neuropathy (DPN) causes morbidity and affects the quality of life. Before diabetes diagnosis, neuropathic damage may be present. Sudoscan provides accurate measurement of the sudomotor function. This study aimed to assess the abnormalities detected by Sudoscan, offered estimates of DPN prevalence, and investigated the relationship between metabolic and clinical parameters. Additionally, we evaluated the diagnostic accuracy of the Sudoscan compared with monofilament and tuning fork tests for detecting DPN.

**Research design and methods:**

Cross-sectional descriptive study including patients with type 2 diabetes for <5 years since diagnosis. We investigated the presence of DPN using a 128 Hz tuning fork test, the 10 g monofilament, and the sudomotor dysfunction in feet using Sudoscan. We compared patients with and without alterations in the Sudoscan. A logistic regression model analyzed variables independently associated with sudomotor dysfunction.

**Results:**

From 2013 to 2020, 2243 patients were included, 55.1% women, age 51.8 years, and 17.1% with normal weight. Monofilament tests and/or tuning fork examination were abnormal in 29% (95% CI 0.23% to 0.27%) and 619 patients (27.6%, 0.25% to 0.29%) had sudomotor alterations. In logistic regression analysis, age ( β =1.01, 0.005–1.02), diastolic blood pressure ( β =0.98, 0.96–0.99), heart rate ( β =1.01, 1.00–1.02), glucose ( β =1.00, 1.00–1.03), albuminuria ( β =1.001, 1.000–1.001), beta-blockers=1.98, 1.21–3.24) and fibrate use=0.61, 0.43–0.87) were associated with sudomotor dysfunction. The AUC (area under the curve) for Sudoscan was 0.495 (0.469–0.522), with sensitivity and specificity of 24% and 71%, respectively.

**Conclusion:**

The Sudoscan identified an important proportion of patients with dysfunction, allowing prompt intervention to decrease the risk for complications.

**Trial registration number:**

NCT02836808.

**WHAT IS ALREADY KNOWN ON THIS TOPIC**

- Diabetic peripheral neuropathy (DPN) affects a high proportion of patients with diabetes, but little is known about its prevalence in patients with recently diagnosed type 2 diabetes.

**WHAT THIS STUDY ADDS**

- Sudomotor dysfunction is found in one-third of patients with a recent diagnosis of type 2 diabetes.
- Evaluation of small fiber function detects almost the same proportion of patients with alterations compared with monofilament and tuning fork tests.
- Older age, lower diastolic blood pressure, heart rate, higher glucose level, albuminuria, and use of beta-blockers and fibrates were associated with sudomotor dysfunction.

**HOW THIS STUDY MIGHT AFFECT RESEARCH, PRACTICE OR POLICY**

- Identification of DPN is essential since diagnosis and certain characteristics of the patients should make awareness of sudomotor dysfunction.

## Introduction

Diabetic neuropathy is a major cause of morbidity and affects the quality of life. Neuropathic symptoms such as burning, pain, and numbness are reported in 42% of patients with diabetes.^1^ Relentless complications such as foot ulcers and amputations result from the interaction between neuropathy and peripheral vascular disease. Neuropathic damage may occur early, even before overt diabetes is diagnosed.^2^ Early neuropathy detection offers a window opportunity for the prevention of these complications.

Sudomotor dysfunction, which may manifest as hypohidrosis/anhidrosis, particularly in hands and feet, represents an early sign of neuropathy. The development of novel techniques such as the Sudoscan, a non-invasive and easy-to-perform test, allows accurate evaluation of sweat gland function based on chloride concentrations through reverse iontophoresis and chronoamperometry.^3^ The Sudoscan has a sensitivity of 75% and specificity of 100%, equivalent to or better than clinical scales for neuropathy identification.^4–6^

We hypothesize that a significant proportion of patients recently diagnosed with type 2 diabetes will have altered sudomotor function not identified with conventional clinical tests. Therefore, we evaluated sudomotor function through electrochemical skin conductance (ESC) in feet using Sudoscan in a cohort of 2243 patients recently diagnosed with type 2 diabetes. We describe the abnormalities detected by Sudoscan, provide estimates of the prevalence of diabetic neuropathy using this method, and explore its association with metabolic and clinical parameters. Additionally, we evaluated the diagnostic accuracy of the Sudoscan in comparison to monofilament and tuning fork tests for detecting DPN.

## Subjects, materials and methods

This is a cross-sectional descriptive study of a cohort of patients less than 5 years since type 2 diabetes diagnosis. We report data from the baseline assessment of 2234 consecutive patients attending the Center of Comprehensive Care for the Patient with Diabetes (“Centro de Atención Integral del Paciente con Diabetes”—CAIPaDi—for its acronym in Spanish).

The CAIPaDi program has been previously described.^7 8^ The CAIPaDi model was approved by the Institutional Ethics and Research Committees (Ref. 1198) and registered on ClinicalTrials.gov (NCT02836808). Informed consent was obtained and signed by all patients. The program includes patients with type 2 diabetes <5 years since diagnosis, 18–70 years old, non-smokers, and without disabling complications. This model seeks to prevent chronic complications of diabetes. Since smoking is a risk factor that needs to be controlled to avoid complications, it was considered an essential requirement that the patients not smoke. However, when a patient requested care at CAIPaDi, and they were smokers, they were referred to the Smoking Clinic and could start their visits after 6 months of having stopped smoking. The diagnosis of diabetes was made according to the American Diabetes Association (ADA) criteria: a random blood glucose concentration >200 mg/dL, fasting glucose >126 mg/dL, glycated hemoglobin (HbA1c) >6.5%, or glucose at 2 hours after a 75 g glucose load >200 mg/dL. Blood tests for fasting glucose, creatinine, lipid profile, urinary albumin/creatinine ratio (ACR) using colorimetric methods (SYNCHRON CX System) and HbA1c using the HPLC method (Bio-Rad Variant II Turbo HbA1c Kit 2) were performed. The laboratory is certified by ISO 90001:2015, the National Glycohemoglobin Standardization Program and the College of American Pathologists. In addition, body composition was assessed by bioimpedance (body composition analyzer JAWON medical ioi353).

A physical assessment was performed on both feet. Plantar hyperkeratosis was classified as present or absent. We used a 128 Hz tuning fork test for the neurological evaluation and the 10 g Semmes-Weinstein monofilament to confirm the perception of vibration and loss of protective sensation (LPS), respectively. The monofilament was applied on 10 sites: 9 on the plantar surface of the foot and 1 on the dorsum. The nine plantar sites were the pulps of the first, third, and fifth toes, over the skin over the first, third, and fifth metatarsal heads, and two in the foot and the heel arch. The dorsal site was in the first web space. Any area with callus was avoided. LPS was considered when the patient did not perceive the stimulus in >4 points. The tuning fork was applied on the distal phalange of the great toe. The patients described when they first noticed the vibration and stopped feeling it. The normal cut-off applied was feeling the vibration for ≥8 s. Diabetic neuropathy was assumed when either one or both tests were abnormal.^9^

A vascular assessment was carried out, identifying tibial, pedal, and popliteal pulses and measuring the ankle-brachial index (ABI). Peripheral arterial disease (PAD) was considered with an ABI <0.9 or>1.3 mm Hg.^10^

Sudomotor function was assessed by Sudoscan (Impeto Medical, Paris, France).^6^ The equipment consists of two sets of stainless steel electrodes for the hands and feet (the sweat glands are densely concentrated in the palms and soles) connected to a computer to record and handle data. The electrodes are used alternately as an anode or cathode, and an incremental direct current of 4 mV is applied to the anode. The device generates voltage toward the cathode through reverse iontophoresis and a current (intensity around 0.2 mA) between the anode and the cathode proportional to the chloride concentration. At low voltages (<10 mV), the stratum corneum is electrically insulating, and only the ducts of the sweat glands have conduction. The electrochemical conduction of the skin, expressed in micro-Siemens (μS), is the ratio between the generated current and the stimulus constant of the direct current (<4 mV) applied to the electrodes. Sudomotor dysfunction was evaluated according to the measurement of the ESC in the feet, according to previous studies: >70 μS=no dysfunction, 70–50 μS=moderate dysfunction, and <50 μS=severe dysfunction.^11–14^

For this analysis, we classified the population into two groups: those with or without Sudoscan alteration (ESC ≤70) in feet. Logistic regression analysis considered sudomotor dysfunction by the Sudoscan as a dependent variable. Covariables were sex, age, diastolic blood pressure (DBP), heart rate (HR), glucose, creatinine, ACR, uric acid, calcium channel blockers, beta-blockers, and fibrate use.

### Statistical analysis

Categorical data are expressed as percentages and compared by Fisher’s exact test. Continuous variables with parametric distribution are expressed as mean±SD, while the non-parametric distribution variables are expressed as median (IQR). We used Pearson correlation to associate metabolic and clinical parameters with diabetic neuropathy. We used logistic regression analyses to assess independent factors associated with the presence of sudomotor dysfunction with the Sudoscan evaluation. We calculated the area under the receiver operating curve to estimate the sensitivity and specificity of the Sudoscan versus the clinical tests.

## Results

A total of 2619 patients with a recent diagnosis of type 2 diabetes attended CAIPaDi from 2013 to 2020, and 2243 had Sudoscan evaluation on their first visit; 55.1% were women, and the mean age was 51.8±10 years. Time since diabetes diagnosis was less than 1 year in 889 (39.6%) patients, 1 year in 297 (13.2%), 2 years in 319 (14.2%), 3 years in 362 (16.1%), 4 years in 293 (13.1%), and 5 years in 71 (3.2%), 384 patients (17.1%) had normal weight, 907 (40.4%) overweight, and 947 patients (42.2%) had obesity. Twenty-two patients (1%) consumed >10 g of alcohol per week.

At the baseline visit, treatment consisted of metformin in 1755 patients (78.2%), sulfonylureas in 464 (20.7%), DPP4 inhibitors in 320 patients (14.3%), basal insulin and/or preprandial boluses in 169 patients (7.5%). Antihypertensive drug use included 298 patients (13.3%) using angiotensin-II receptor antagonists and 154 (6.9%) ACE inhibitors. These were followed by diuretics used in 154 patients (6.9%). Lipid-lowering medications included 315 patients (14%) on fibrates and 238 (10.6%) statins. Additionally, 195 patients (8.7%) used acetylsalicylic acid for the primary prevention of cardiovascular diseases.

A neurological examination showed LPS in 55 patients (2.5%) and altered vibration sensation with a tuning fork in 503 patients (22.4%). Adding patients with any abnormality (monofilament or tuning fork), the prevalence of neuropathy was 29%, 95% CI 0.23% to 0.27%.

The popliteal pulse was absent in 3 patients (0.1%), tibial pulse in 5 patients (0.2%), and pedal pulse in 15 patients (0.7%). In 72 patients (3.2%, 95% CI 0.025% to 0.040%), we found an ABI <0.9, 2117 patients (94.7%, 95% CI 0.93% to 0.95%) had ABI 0.9–1.3, and 47 patients (2.1%, 95% CI 0.015% to 0.028%) had ABI >1.3.

### Abnormalities detected by Sudoscan

The median ESC in both feet was 69 μS (60–76), with an asymmetry of 3% (1%–6%) consistent with the predominantly symmetric pattern of distal neuropathy. A total of 619 patients (27.6%, 95% CI 0.25% to 0.29%) had sudomotor alteration in feet.

In the group with sudomotor dysfunction was a higher prevalence of women. Individuals in this group were older by approximately 1 year and had lower DBP, higher HR, higher glucose, higher ACR in those with dysfunction, and lower uric acid (table 1). A higher proportion of individuals with sudomotor dysfunction used calcium channel blockers and beta-blockers, and a lower proportion used fibrates. Online supplemental table 1 shows the patients’ hypoglycemic, antihypertensive, and lipid-lowering drugs according to sudomotor dysfunction.

10.1136/bmjdrc-2022-003005.supp1 Supplementary data

**Table 1:** Characteristics of the population according to dysfunction in feet identified by the Sudoscan

	All (N=2243)	Without dysfunction (N=1620)	Dysfunction (N=619)	P value
Women	1236 (55.2%)	850 (52.5)	386 (62.4)	<0.01
Age, years	52±10	51.4±10.1	52.8±10.2	0.004
Time since diagnosis (years)				0.86
0	889 (39.8)	653 (40.5)	236 (38.2)	
1	297 (13.3)	212 (13.1)	85 (13.1)	
2	319 (14.3)	229 (14.2)	90 (14.6)	
3	362 (16.1)	264 (16.4)	98 (15.9)	
4	293 (13.1)	208 (12.9)	85 (13.8)	
5	71 (3.2)	48 (3.0)	23 (3.7)	
Systolic blood pressure (mm Hg)	125±15.5	125±15	124±16	0.33
Diastolic blood pressure (mm Hg)	77±7.7	77±7	76±7	0.04
Heart rate (BPM)	63±11	75±11	76±11	0.01
Triglycerides (mg/dL)	171 (123–241)	171 (123–240)	171 (125–243)	0.70
Total cholesterol (mg/dL)	189±43	188±43	191±42	0.16
HDL-cholesterol (mg/dL)	43±10	43±10	44±10	0.11
LDL-cholesterol (mg/dL)	115±36	114±35	116±37	0.42
Non-HDL cholesterol (mg/dL)	145±42	145±42	147±41	0.30
Glucose (mg/dL)	130 (110–183)	128 (105–181)	137 (107–194)	0.05
HbA1c (%)	8.4±2.4	8.3±2	8.5±2	0.17
Creatinine (mg/dL)	0.7±0.17	0.7±0.1	0.7±0.1	0.01
Albumin/creatinine ratio (mg/g)	8.9 (4.9–20.9)	8.6 (4.7–19.3)	9.6 (5.3–25.1)	0.004
Albumin/creatinine ratio >30 mg/g (%)	394 (17.6)	259 (16.2)	135 (22.3)	<0.01
Uric acid (mg/dL)	5.3±1.4	5.3±1.4	5.2±1.4	0.03
Body mass index (kg/m^2^)	29.6±5	29.5±4.8	29.8±5.3	0.12

Data expressed as number and percentage, mean±SD or median (IQR). Dysfunction was considered as ESC ≤70 ESC in the foot evaluation.

BPM, beats per minute; ESC, electrochemical skin conductance; HbA1c, glycated hemoglobin; HDL-cholesterol, high density lipoprotein cholesterol; LDL-cholesterol, low density lipoprotein cholesterol.

In the logistic regression analysis (table 2), higher age, lower DBP, higher HR, glucose, ACR, and beta-blocker use were associated with sudomotor dysfunction. In contrast, treatment with fibrates was associated with no dysfunction detected by the Sudoscan. The model explained 4.8% of the variation of the sudomotor dysfunction (Hosmer-Lemeshow test p=0.447).

**Table 2:** Logistic regression analysis

Variable	Wald	P value	B	95% CI
Sex	3.494	0.062	1.266	0.98 to 1.62
Age	8.619	0.003	1.015	0.005 to 1.026
DBP	9.128	0.003	0.980	0.967 to 0.993
Heart rate	8.120	0.004	1.013	1.004 to 1.022
Glucose	4.397	0.036	1.002	1.000 to 1.003
Creatinine	1.337	0.248	0.641	0.302 to 1.362
ACR	7.175	0.007	1.001	1.000 to 1.001
Uric acid	0.006	0.938	0.997	0.920 to 1.080
Calcium channel blockers	2.776	0.096	1.414	0.941 to 2.126
Beta-blockers	7.432	0.006	1.982	1.212 to 3.241
Fibrates	7.501	0.006	0.615	0.435 to 0.871

r^2^: 4%; Hosmer-Lemeshow test p=0.447.

ACR, albumin/creatinine ratio; DBP, diastolic blood pressure.

### Diagnostic accuracy of the Sudoscan

Sudoscan detected 619 patients (27.6%) with sudomotor dysfunction, while 650 patients (28.9%) had DPN identified using a tuning fork or monofilament. Only 180 patients (8.0%) had abnormal results with the Sudoscan and the clinical tests. The Sudoscan has an area under the curve (AUC) of 0.495 (IC 95% 0.469–0.522) with a sensitivity of 24% and specificity of 71% for detecting neuropathy.

## Discussion

We evaluated sudomotor dysfunction in a cohort of patients with a recent diagnosis of type 2 diabetes. The results show that the prevalence of dysfunction evaluated with the Sudoscan is 27.6% compared with 29% of neuropathy identified using conventional tests. The low asymmetry (3%) of sudomotor dysfunction found supports the diagnosis of diabetic neuropathy.^15^ Additionally, the proportion of patients with vascular alterations was low (<1%).

The ADA indicates that all patients with type 2 diabetes should have a screening for peripheral neuropathy at diagnosis and at least once a year. Early neuropathy can be asymptomatic, increasing the risk of developing foot complications. We found an important proportion of individuals with alterations even when the time since diagnosis was <5 years. These findings indicate that diabetes diagnosis might be late or other alterations, such as pre-diabetes and obesity, might also be related to neuropathy.^16^ As there is no treatment for neuropathy, early identification and awareness of alterations should be made to reduce or delay chronic complications with appropriate interventions.^9^ In addition, an early diabetes diagnosis is imperative to decrease related complications.

The Sudoscan has been validated for the identification of small fiber neuropathy. Small fiber dysfunction usually presents before large fiber alterations and represents the earliest sign of diabetic neuropathy.^17–19^ Therefore, its use allows the detection of early changes that are not evident with conventional tests. In a study analyzing data from 144 patients with type 2 diabetes, peripheral neuropathy was identified in 27.8%. The authors estimated that the best cut-off of feet ESC to detect diabetic neuropathy was 54 µS.^20^ In another study, including Mexican individuals, patients with worse electroconductance had a more severe neuropathy (according to Michigan Neuropathy Screening Instrument), were older, and had lower BMI.^21^ In this study, the Sudoscan did not identify a higher proportion of individuals affected. In addition, only a small proportion of the individuals were identified with alterations using conventional tests and the Sudoscan. A possible explanation is that the tests identify different alterations (individuals with early vs more advanced DPN) and the participants had <5 years since diabetes diagnosis, although we expected that the Sudoscan identified a higher proportion of individuals with Sudomotor dysfunction.

Diverse factors have been associated with peripheral neuropathy. In a meta-analysis that included 16 studies with 12 116 participants, the duration of diabetes, age, HbA1c, and diabetic retinopathy were associated with increased risk for diabetic neuropathy.^22^ In a study including Mexican individuals, the risk factors associated with diabetic neuropathy were diabetes duration, glycemic exposure index, low-density and high-density lipoprotein levels, metformin treatment, diabetic retinopathy, and smoking.^23^ There were significant differences among variables in the group with and without dysfunction detected by the Sudoscan. After adjustment, DBP, HR, glucose, ACR, use of beta-blockers, and fibrates remained significantly associated with sudomotor dysfunction in this cohort of individuals with recent type 2 diabetes diagnosis. Beta-blockers have no direct impact on sudomotor function. However, the use of these medications might indicate patients with greater blood pressure or a longer development of the condition. It is not unexpected that individuals using these drugs exhibit worse sudomotor function since more comorbidities are expected in these patients.^24^ This emphasizes that these variables should be considered in patients with diabetes regardless of the time since diagnosis.

Important strengths of this work should be acknowledged. We describe the prevalence of abnormalities in patients with a recent diagnosis of diabetes detected by Sudoscan. Another strength is the large number of patients evaluated with a complete clinical and biochemical evaluation. Limitations of this study include the cross-sectional design and the lack of a control group without diabetes. In a small proportion of individuals included in the program (14.3%), the sudomotor evaluation is not available because, initially, this test was not included in the evaluations performed. We did not perform an extensive evaluation to exclude other etiologies of sudomotor dysfunction. Finally, it would have been desirable to measure small fiber function.

The Sudoscan is a useful diagnostic tool that helps to identify early neuropathy. This study identified an important proportion of patients with dysfunction, allowing prompt intervention to decrease the risk of complications.

## Data Availability

Data are available on reasonable request.
