# Supplementary material for: Detection of sudomotor alterations evaluated by Sudoscan in patients with recently diagnosed type 2 diabetes
Source: BMJ Open Diabetes Res Care. 2022 Dec 15;10(6):e003005. doi: 10.1136/bmjdrc-2022-003005 (PMC9756300; doi:10.1136/bmjdrc-2022-003005)
Supplement: Supplementary data [file bmjdrc-2022-003005supp001.pdf]

Supplementary Table 1. Treatment of the participants

Hypoglycemic agents	All	Without dysfunction (N= 1620)	Dysfunction (N= 619)	p
Metformin (%)	1755 (78.2)	1263 (77.8)	492 (79.5)	0.38
SGLT-2 inhibitors (%)	74 (3.3)	54 (3.3)	20 (3.2)	0.91
Sulphonylureas (%)	464 (20.7)	340 (20.9)	124 (20.0)	0.63
GLP1 agonists (%)	7 (0.3)	7 (0.4)	0 (0.0)	0.10
DPP-4 inhibitors (%)	320 (14.3)	244 (15.0)	76 (12.3)	0.09
Thiazolidinedione (%)	43 (1.9)	31 (1.9)	12 (1.9)	0.96
Basal insulin (%)	169 (7.5)	95 (5.8)	32 (5.2)	0.53
Preprandial insulin (%)	21 (0.94)	16 (1.0)	5 (0.8)	0.69
Antihypertensives				
Angiotensin-converting enzyme inhibitors (%)	154 (6.9)	115 (7.1)	39 (6.3)	0.51
Calcium channel blockers (%)	119 (5.3)	76 (4.7)	43 (6.9)	0.03
Beta-blockers (%)	73 (3.3)	41 (2.5)	32 (5.2)	0.002
Angiotensin-II receptor antagonist (%)	298 (13.3)	212 (13.1)	86 (13.9)	0.60
Diuretic	154 (6.9)	109 (6.7)	45 (7.3)	0.64
Lipid-lowering				
Statins (%)	315 (14)	233 (14.3)	82 (13.2)	0.50
Fibrates (%)	238 (10.6)	192 (11.8)	46 (7.4)	0.03
Ezetimibe (%)	13 (0.57)	10 (0.6)	3 (0.5)	0.71
Aspirin (%)	195 (8.7)	146 (9.0)	49 (7.9)	0.42
Alpurinol (%)	17 (1.0)	14 (1.0)	3 (0.5)	0.20

SGLT-2 inhibitors: Sodium-glucose Cotransporter-2; GLP1 agonists: Glucagon-like Peptide-1 Receptor Agonists; DPP-4 inhibitors: Dipeptidyl Peptidase IV
